# Particulate matter and atherosclerosis: a bibliometric analysis of original research articles published in 1973–2014

**DOI:** 10.1186/s12889-016-3015-z

**Published:** 2016-04-19

**Authors:** Feifei Wang, Xiaofeng Jia, Xianliang Wang, Yongdong Zhao, Weidong Hao

**Affiliations:** Department of Toxicology, School of Public Health, Peking University, Beijing, 100191 China; State Key Laboratory of Environmental Criteria and Risk Assessment, Chinese Research Academy of Environmental Sciences, Beijing, 100012 China; Institute of Medical Information & Library, Chinese Academy of Medical Sciences and Peking Union Medical College (CAMS & PUMC), Beijing, 100020 China; Department of Toxicology, Baotou Medical College of Public Health, Baotou, 014040 China

**Keywords:** Bibliometric analysis, Particulate matter, Atherosclerosis, Research tendency, Popular topics

## Abstract

**Background:**

Epidemiological and experimental studies have suggested that exposure to particulate air pollution may promote progression of atherosclerosis.

**Methods:**

In the present study, the characteristics and trends of the research field of particulate matter (PM) and atherosclerosis were analyzed using bibliometric indicators. Bibliometric analysis was based on original papers obtained from PubMed/MEDLINE search results (from 1973 to 2014) using Medical Subject Headings (MeSH) terms. A fully-detailed search strategy was employed, and articles were imported into the Thomson Data Analyzer (TDA) software.

**Results:**

The visualizing network of the collaborative researchers was analyzed by Ucinet 6 software. Main research topics and future focuses were explored by co-word and cluster analysis. The characteristics of these research articles were summarized. The number of published articles has increased from five for the period 1973–1978 to 89 for the period 2009–2014. Tobacco smoke pollution, smoke and air PM were the most studied targets in this research field. Coronary disease was the top health outcome posed by PM exposure. The aorta and endothelium vascular were the principal locations of atherosclerotic lesions, which were enhanced by PM exposure. Oxidative stress and inflammation were of special concern in the current mechanistic research system. The top high-frequency MeSH terms were clustered, and four popular topics were further presented.

**Conclusion:**

Based on the quantitative analysis of bibliographic information and MeSH terms, we were able to define the study characteristics and popular topics in the field of PM and atherosclerosis. Our analysis would provide a comprehensive background reference for researchers in this field of study.

## Background

Exposure to air particulate matter (PM) may result in various diseases including cardiovascular diseases (CVD) such as atherosclerosis, myocardial infarction, stroke, myocardial ischemia, coronary diseases and cardiac arrhythmia. Its etiology and pathogenic mechanisms may be complex, which include pulmonary and systemic inflammation, accelerated atherosclerosis and altered cardiac autonomic functions [1, 2]. PM contributes to increased cardiac risk by initiating and promoting atherosclerotic progression, which is the underlying cause of most cardiovascular diseases [3]. There is ample epidemiological, clinical and experimental evidence supporting the association of PM with atherogenesis [4–7]. Related research on PM exposure-enhanced atherosclerosis has attracted greater attention and is becoming an important, rapid-progressing field in the coming years. Therefore, it is necessary to survey bibliometric characteristics and portray the overall trend of these research fields.

Bibliometrics refers to a research methodology employed in library and information sciences, which utilizes quantitative analysis and statistics to describe the bibliographic information of articles (year publication, title, authors, publisher, affiliations, etc.) within a given topic, field, institution, and/or country [1, 8]. Recently, bibliometrics has been used to explore trends in biomedical, medical and environmental research fields; such as the mapping of global drinking water from 1992 to 2011 [8], air PM on the cardiovascular system [1], urban health for the period 1978–2012 [1], stem cell research in Iran [9], cardiovascular research from Latin America from 1999 to 2008 [10], and ophthalmology research from 1997 to 2009 [11]. Bibliometrics applies quantitative and statistical methods to analyze a variety of phenomena across scientific literatures. Literature data are mainly derived from international literature databases such as PubMed/MEDLINE [1], Springerlink [12], and Web of Science [13]. In the present study, we attempted to analyze the characteristics and trends within the research fields of PM and atherosclerosis using the bibliometric approach by conducting a search on the PubMed/MEDLINE database using Medical Subject Headings (MeSH) terms.

## Methods

### Data sources

The database used in the present study was PubMed/MEDLINE, which is provided by the National Library of Medicine (NLM) in America. The NLM covers the largest number of publications on life sciences and biomedical research.

### Search strategy

MeSH, compiled by NLM for its bibliographies and cataloging [14], were used to perform the literature search and exploration of popular topics in these research fields. As illustrated in Table 1, these MeSH terms (2015 MeSH) were combined using the Boolean operator AND. The first group of MeSH terms included PM and the expanded versions of the MeSH term such as particle size, air pollution and air pollutants. The second group of MeSH terms included atherosclerosis and the expanded versions of the MeSH of arteriosclerosis, atherosclerotic plaque, carotid artery diseases and coronary diseases.

**Table 1:** Reference search strategy

Connector	Field	Parameter
	Mesh Terms	(Particulate Matter) OR ((Air Pollution) OR (Air Pollutants) AND (Particle Size))
AND	Mesh Terms	(Atherosclerosis) OR (Arteriosclerosis) OR (Coronary Disease) OR (Plaque, Atherosclerotic) OR (Carotid Artery Diseases)
AND	Publication type	Journal Article
NOT	Publication type	Review
AND	Language	English

### Data collection

Data was obtained through a comprehensive literature search of the PubMed/MEDLINE database on the 8th of January, 2015. The eligible records of 259 articles were downloaded. Bibliographic information included the title, abstract, name of authors, journal title, year of publication, and the corresponding authors’ address.

### Data analysis

These records were imported into the Thomson Data Analyzer software version 3.0 (TDA; Thomson Reuters Co., New York, NY, USA), which could provide a global view of the technology area. In order to analyze characteristics and trends, the data analysis process consisted of five parts: data cleaning, bibliographic information analysis, MeSH terms analysis, co-occurrence of word (co-word) analysis, and cluster analysis. In order to ensure the accuracy of these results, reduplicates and conflicting data were cleaned using the TDA software, which offers an efficient and accurate automatic data cleansing tool. For subsequent analysis, a bibliographic information database with 259 articles and 683 MeSH terms used during the period 1973–2014 was established. TDA was used to process and analyze bibliographic information (year of publication, journal title, affiliation, author and country).

The visualized network of the collaborative researchers was analyzed by Ucinet 6 (Analytic Technologies Co., Lexington, KY, USA) software, which is considered the most popular social network analysis software and features a strong matrix analysis function. The 50 × 50 co-occurrence frequency matrix of the 50 most published authors was calculated to reflect the collaborative relationship between top researchers. The betweenness centrality for measures of structural centrality in social networks can reflect the degree of centralization of the research network [15]. MeSH terms were ranked according to frequency. The top 225 MeSH terms (with a cumulative frequency of 74.2 %) were defined as high-frequency terms for the subsequent research trend analysis.

Co-occurrence of word (co-word) analysis is a basic and important approach for themes exploration, which include three steps: measurement of the links between terms, organization of clusters, and concluding the topics [1]. The 225 × 225 co-occurrence frequency matrices of the top 225 MeSH terms were conducted using the TDA software. After conducting the cluster analysis with SPSS software version 17.0 (Chicago, IL, USA), the MeSH terms were combined to create 12 clusters according to the degree of similarity. Based on the cluster analysis of high-frequency MeSH terms, popular topics in the research fields of PM and atherosclerosis were concluded.

## Results

### Characteristics of publications

#### Publication outputs

**Fig. 1:**
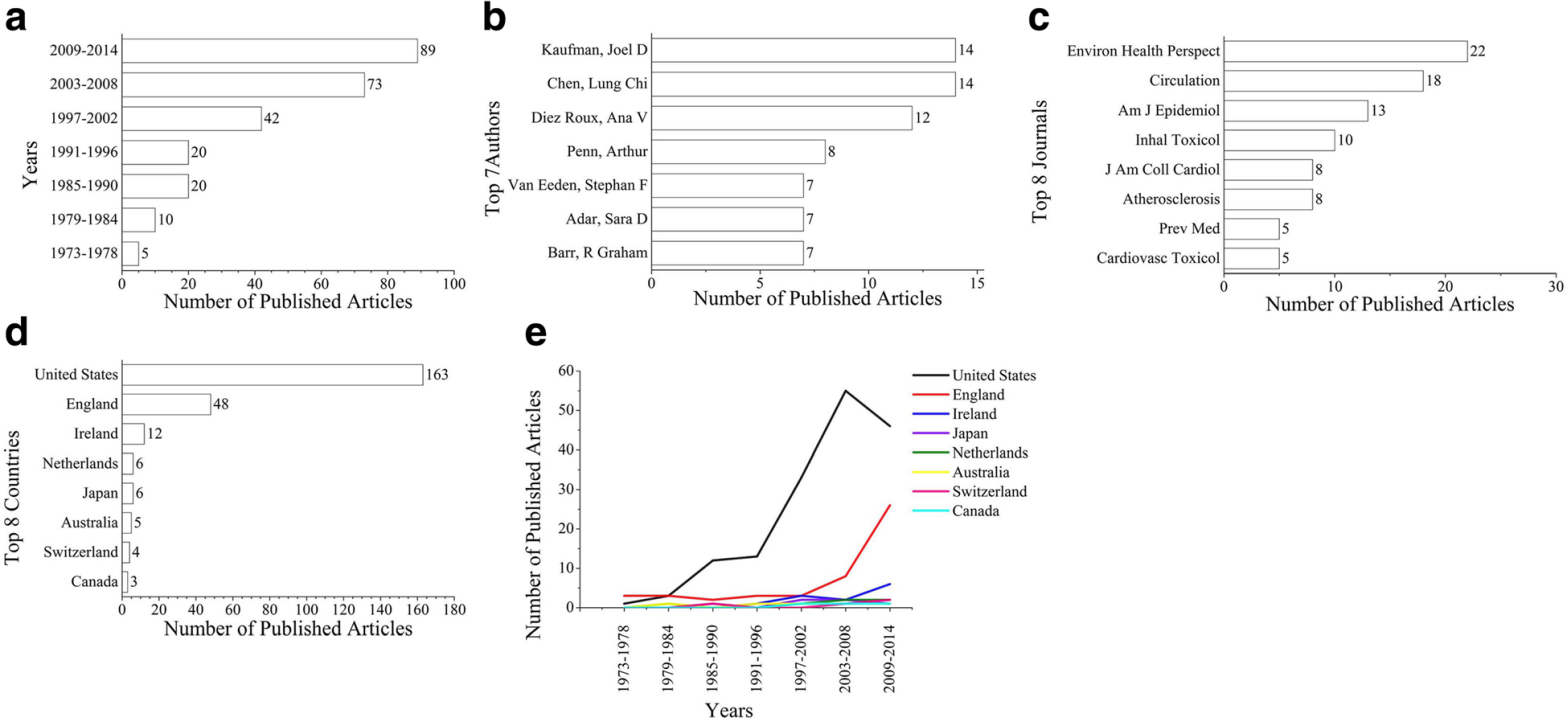
Characteristics of published articles on the study of PM exposure-related arteriosclerosis. The numbers of papers for every 6-year segment from 1973 to 2014 are shown in (**a**). The numbers of papers of the top authors, journals and countries are shown in (**b**), (**c**) and (**d**), respectively. The trends of published articles for the top eight countries during the period 1973–2014 are shown in (**e**)

Due to the large number of publications retrieved, more attention was given to the research area of PM and atherosclerosis. As shown in Fig. 1a, the number of published articles increased from five occurrences for the period 1973–1978 to 89 occurrences for the period 2009–2014. As shown in Fig. 1b, Dr. Kaufman and Dr. Chen LC had authored most of the published articles in the field of PM exposure-related atherosclerosis. As shown in Fig. 1c, journals on Environmental Health Perspectives and Circulation had covered the largest portion of published articles. As shown in Fig. 1d, USA was the top country that conducted related studies. As shown in Fig. 1e, there has been an increasing trend of published articles from other countries.

#### Characteristics of the top fifty researchers in the field of PM exposure-related atherosclerosis

**Fig. 2:**
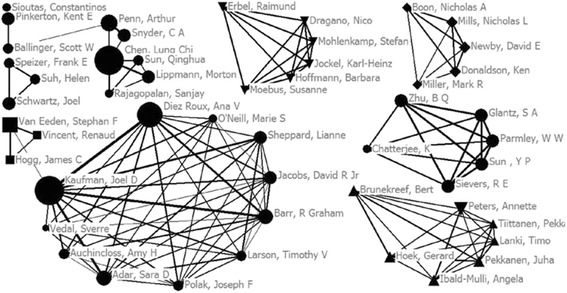
Collaborative relationship network of the top 50 researchers. The size of the nodes represents the number of papers. The size of the lines represents the close degree of co-authors. The shapes of the nodes represent different countries (circle represents the US, down-triangle represents Germany, box represents Canada, up-triangle represents Finland, and diamond represents UK)

As shown in Fig. 2, the research network included USA, Germany, Finland, Canada and UK. Researchers from USA constituted the largest research network. Furthermore, Dr. Kaufman and Dr. Chen LC published most of the articles in the field of PM exposure-related atherosclerosis, but Dr. Kaufman was the central researcher and acted as a bridge that connected the whole network (betweenness centrality = 31).

#### Characteristics of the high-frequency MeSH terms

As shown in Tables 2 and 3, the MeSH terms were ranked according to frequency and were classified into eight categories including PM, objects-human, objects-animals, objects-lesions, main outcomes, mechanisms, main methods and factors. Epidemiological studies, in which ‘middle-aged’ and ‘aged’ populations were the main focus, comprised of a significant proportion of the research field of PM exposure-related atherosclerosis; and were mostly cohort studies. Mice, gene knockout mice and disease model animals were the most frequently chosen subjects in vivo. Oxidative stress and inflammation were the most popular mechanisms hypothesized. The methods of cell culture in vitro were very important in toxicological and mechanistic studies, and endothelial cells were the major experimental model. Tobacco smoke pollution, smoke and air PM were the most studied targets in the research fields. The aorta and endothelium vascular were the principal atherosclerosis lesion locations, which were enhanced by PM exposure. Coronary disease was the top health outcome posed by PM exposure. Time, gender and diet were the top three risk factors that may influence the degree of atherosclerosis accelerated by PM exposure.

**Table 2:** Characteristics of high-frequency MeSH terms

	PM		Objects-humans		Objects-animals		Objects-lesions	
Rank	MeSH terms	Freq.	MeSH terms	Freq.	MeSH terms	Freq.	MeSH terms	Freq.
1	Tobacco Smoke Pollution	149	Humans	192	Animals	80	Aorta	37
2	Smoke	84	Male	186	Mice	45	Endothelium, Vascular	26
3	Particulate Matter	70	Female	150	Mice, Knockout	34	Tunica Intima	12
4	Air Pollutants	50	Middle Aged	108	Disease Models, Animal	20	Carotid Arteries	8
5	Particle Size	37	Aged	88	Rabbits	16	Myocardium	7
6	Air Pollution	33	Adult	87	Rats	8	Brachial Artery	6
7	Vehicle Emissions	16	Aged, 80 and over	22	Chickens	7	Carotid Artery, Common	6
8	Carbon Monoxide	14	Adolescent	16	Dogs	3	Tunica Media	6

**Table 3:** Characteristics of high-frequency MeSH terms

	Main outcomes		Mechanisms		Main methods		Factors	
Rank	MeSH terms	Freq.	MeSH terms	Freq.	MeSH terms	Freq.	MeSH terms	Freq.
1	Coronary Disease	128	Oxidative Stress	19	Cohort Studies	24	Risk	97
2	Atherosclerosis	73	Inflammation	13	Prospective Studies	21	Time Factors	33
3	Arteriosclerosis	52	Vasodilation	11	Cross-Sectional Studies	19	Sex Factors	12
4	Lung Neoplasms	21	Gene Expression Regulation	8	Questionnaires	14	Diet, Atherogenic	11
5	Heart Rate	14	C-Reactive Protein	8	Biological Markers	13	Antioxidants	10
6	Myocardial Infarction	13	Muscle, Smooth, Vascular	8	Follow-Up Studies	13	Vitamin	9
7	Blood Pressure	12	Vasoconstriction	8	Case–control Studies	10	Age Factors	7
8	Cardiovascular Diseases	9	Endothelial Cells	7	Cells, Cultured	10	Life Style	5

### Research tendencies and hotspots

Cluster analysis of the top 225 high-frequency MeSH terms was classified into 10 clusters, which explained 99.05 % of total variations. According to the roles of MeSH terms in the studies and relying on professional judgment, the terms in every cluster were categorized into objects, exposure variables, main outcome variables, factors, lesions, designs/methods and contents/mechanisms. Cluster analysis can group separate data types into clusters, and help identify valuable clues of possible research issues. After the synthetic analysis of the ten clusters, four research hotspots related to PM and atherosclerosis were identified, which provide a reference for further research in these fields (Tables 4, 5, 6 and 7). Results of the cluster analyses reflected the research tendencies in these research fields. Topic 1 was formed by combining Clusters 1, 3, and 9; Cluster 2 was combined with Cluster 4 to form Topic 2; Clusters 5, 7, and 8 were converged to form Topic 3; and Cluster 6 was combined with Cluster 10 to form Topic 4 (Table 8).

**Table 4:** Cluster analysis of MeSH terms (Topic1)

Cluster	MeSH terms
1	Objects: Humans; Male; Female; Middle Aged; Aged; Adult
	Exposure variables: Tobacco Smoke Pollution
	Main outcome variables: Coronary Disease
3	Exposure variables: Smoke
	Main outcome variables: Arteriosclerosis; Aortic Diseases; Heart Diseases; Hypertension; Body Mass Index;
	Contents/Mechanisms: Apolipoprotein; Cholesterol; Antioxidants; Lipid; Lipoproteins LDL; Lipoproteins HDL; Hyperlipidemias;
	Peroxidation; Genetic Predisposition to Disease; Thiobarbituric Acid Reactive Substances; Glutathione; Ascorbic Acid; Vitamin; Chromatography High Pressure Liquid
	Factors: Atherogenic Diet; Workplace; Health Education; Educational Status
9	Objects: Child; Parents/psychology
	Exposure variables: Cotinine
	Designs/Methods: Case–control Studies; Biological Markers
	Contents/Mechanisms: Vasodilation; Endothelium, Vascular; Vasodilator Agents; Nitroglycerin; Brachial Artery

**Table 5:** Cluster analysis of MeSH terms (Topic2)

Cluster	MeSH terms
2	Objects: Aged-80 and over; Ethnic Groups; Continental Population Groups; Europe; USA; Spouses
	Exposure variables: Air Pollutants; Air Pollution; Air Pollution-Indoor; Environmental Exposure; Occupational Exposure; Environmental Monitoring; Soot; Nitrogen Dioxide; Nitrogen Oxides
	Designs/Methods: Epidemiologic Studies; Cohort Studies; Prospective Studies; Cross-Sectional Studies; Follow-Up Studies; Questionnaires; Prevalence; Odds Rati; Proportional Hazards Models; Multivariate Analysis; Logistic Models; Longitudinal Studies; Linear Models; Confidence Intervals
	Main outcome variables: Carotid Intima-Media Thickness; Carotid Artery Diseases; Stroke; Cardiovascular Diseases; Vascular Calcification; Respiratory Tract Diseases; Pulmonary Disease-Chronic Obstructive; Lung Neoplasms; Neoplasms; Occupational Diseases; Sensitivity and Specificity; Tomography X-Ray Computed; Predictive Value of Test; Public Health; Comorbidity; Hospitalization; Cause of Death
	Factors: Time Factors; Sex Factors; Age Factors; Socioeconomic Factors; Life Style; Obesity; Health Behavior; Health Status
4	Lesions: Tunica Intima; Carotid Arteries; Tunica Media

**Table 6:** Clusters analysis of MeSH terms (Topic3)

Cluster	MeSH terms
6	Objects: Infant; Pregnancy; Adolescent; Aging; European Continental Ancestry Group; Rats; Rabbits; Chickens; Dogs; Great Britain; Africa; Canada/epidemiology
	Exposure variables: Carbon Monoxide; Dust; Nitric Oxide; Sulfur Dioxide; Metals; Nanoparticles; Tars; Urban Health; Fires
	Designs/Methods: Retrospective Studies; Random Allocation; Single-Blind Method; Confounding Factors (Epidemiology); Marriage; Residence Characteristics; Models-Statistical; Regression Analysis; Analysis of Variance; Blood Chemical Analysis; Respiratory Function Tests; In Vitro Techniques
	Contents/Mechanisms: Inflammation; Muscle- Smooth-Vascular; Cytokines; Interleukin-6/metabolism; Carboxyhemoglobin; Immunohistochemistry; Platelet Aggregation/drug effects; Reactive Oxygen Species; Endothelin-1; Cell Movement; Tumor Necrosis Factor-alpha; Vascular Cell Adhesion Molecule-1/metabolism; Lipid Metabolism; Interleukin-1beta; Apoptosis; Myocytes-Smooth Muscle Leukocyte Count; Lipids/blood; Receptors-LDL; Fibrinogen; Aspirin; Temperature; Cyclooxygenase 2; Neutrophils; Macrophages/metabolism; Monocytes; Bronchoalveolar Lavage Fluid
	Main outcome variables: Cerebrovascular Disorders; Asthma; Lung Diseases; Diabetes Mellitus; Acute Disease; Disease Progression; Blood Pressure; Pulmonary Artery; Carotid Artery-Common; Blood Vessels; Brachiocephalic Trunk; Endothelins Arteries; Lung; Heart; Liver; Pneumonia; Intermittent Claudication; Mortality; Prenatal Exposure Delayed Effects
	Exposure variables: Particulate Matter; Particle Size; Inhalation Exposure; Vehicle Emissions; China
10	
	Designs/Methods: Cross-Over Studies; Double-Blind Method; Electrocardiography
	Contents/Mechanisms: Vasomotor System; Cardiovascular System; C-Reactive Protein/analysis;
	Main outcome variables: Myocardial Infarction; Myocardial Ischemia; Thrombosis; Heart Rate; Arrhythmias-Cardiac

**Table 7:** Cluster analysis of MeSH terms (Topic4)

Cluster	MeSH terms
5	Objects: Endothelial Cells; Cell Line
	Study designs and methods: Cells Cultured; Dose–response Relationship-drug; Microscopy
	Contents/Mechanisms: RNA Messenger; Gene Expression; Gene Expression Regulation; Up-Regulation; Antigens-CD; Heme Oxygenase-1, Tyrosine
	Main outcome variables: Vasoconstriction; Myocardium
7	Contents/Mechanisms: Oxidative Stress; Superoxide Dismutase,Oxidants; Mitochondria; DNA-Mitochondrial, DNA Damage
8	Objects: Animals; Mice; Mice-Knockout; Disease Models- Animal
	Contents/Mechanisms: Aorta; Apolipoproteins E
	Main outcome variables: Atherosclerosis

**Table 8:** Four high-frequent topics in the research of particulate matter and atherosclerosis

Topics	Cluster	Explained variation, %
Topic 1. Epidemiological studies on the associations between tobacco exposure and atherosclerosis	Clusters 1, 3, and 9	3.5, 10.67, 4.44
Topic 2. Epidemiological studies on the associations between environmental particulate matter exposure and atherosclerosis	Clusters 2 and 4	24.44, 1.33
Topic 3. studies on the different roles of particulate matter size/components/source in health effects	Clusters 6 and 10	35.11, 7.11
Topic 4. Toxicological studies on particulate matter-induced arteriosclerosis	Clusters 5, 7, and 8	6.67, 2.67, 3.11

## Discussion

Topic 1 (Clusters 1, 3, and 9) included epidemiological studies on the associations between tobacco exposure and atherosclerosis. It is well-known that smoking is an important source of indoor air PM pollution [16] and levels of PM_2.5_ is a marker for secondhand tobacco smoke (SHS) [17]. Both active smoking and exposure to environmental tobacco smoke (ETS) were associated with the progression of atherosclerosis [18, 19]. Cigarette smoking is a powerful risk factor for atherosclerotic diseases in middle-aged and aged populations [20–22]. Moreover, smoking is associated with lipoprotein abnormalities, which promote arterial lipid accumulation and atherogenesis [23–26]. SHS exposure or the inhalation of ETS is an independent risk factor for subclinical coronary atherosclerosis, coronary heart disease (CHD) and peripheral arterial disease (PAD). In addition, long-term exposure to SHS creates an imbalance in the lipid profile. This imbalance leads to lipid accumulation in the blood vessels of the heart and aorta in experimental models of human conditions in vivo, which in turn leads to atherosclerotic plaque formation [27]. In this topic, the association between familial and fetal tobacco smoke exposures and vascular damage also received close attention, because young adults/children/infants are at high risk of SHS exposure (Cluster 9). These studies show that thicker common carotid artery intima-media thickness (CIMT) in young adulthood is associated with fetal tobacco smoke exposure [28–30]. Exposure to ETS is independently associated with decreased brachial artery flow-mediated dilation, decreased aortic elastic properties and increased ApoB levels among adolescents with increased cotinine level (a sensitive and specific biomarker of ETS exposure) [29, 31, 32]. Moreover, smoke-free legislation is associated with lower risk of smoking-related cardiac and cerebrovascular diseases [33–35].

Topic 2 (Clusters 2 and 4) covered epidemiological studies on the associations between environmental PM exposures and atherosclerosis. Epidemiological findings show a clear association between long-term exposure to PM_2.5_/PM_10_ and atherosclerosis. The studies reviewed on this topic focused on the effects of PM air pollution on subclinical atherosclerosis by means of noninvasive imaging (a predictor of cardiovascular events). Long-term PM concentrations are associated with increased CIMT progression [36–39], but does not appear to be associated with greater arterial stiffness [40]. CIMT, a marker of subclinical atherosclerosis, is associated with atherosclerosis development and progression in middle-aged populations [41]. The investigation of links between PM exposures and subclinical atherosclerosis mostly adopts the cohort and cross-sectional studies. The most studied cohort is the Multi-Ethnic Study of Atherosclerosis (MESA) conducted across six study sites in the USA [36, 38, 40, 42]. In addition, exposure to traffic is also associated with atherosclerosis [43].

Topic 3 (Clusters 6 and 10) included studies on the different roles of PM components/sources in health effects. On this topic, the source that has the most concern is vehicle emissions (Cluster 10), which is linked with heart rate variability (HRV) [44], cardiac repolarization [45] and endothelial gene regulation [46]. The sources of PM from fire/combustion emission are also issues of concern. Exposure to ultrafine particles during fire suppression is considered a potential contributing factor for CHD [47]. Combustion emissions cause pro-atherosclerotic responses in ApoE^−/−^ mice [48]. Moreover, these components (carbon black, metals, etc.) deserve closer attention. Nano-sized carbon black particles are associated with modest vasomotor impairment [49]. The zinc metal component of PM drives cardiovascular health effects, as well as the possible susceptibility induced by hyperlipidemia [50]. In addition, other air pollutants (nitrogen dioxide, sulfur dioxide, carbon monoxide, etc.) may also induce cardiovascular risk on their own, or combined with PM exposure. Elevated concentrations of PM and nitrogen dioxide increase the risk of supraventricular runs, as well as the number of ventricular runs [51].

Topic 4 (Clusters 5, 7, and 8) included toxicological studies on PM-induced arteriosclerosis. Mice, mice-knockout and disease model animals were chosen as study subjects for animal experiments (Cluster 8). ApoE^−/−^ mice, which is probably of more concern, lacks apolipoprotein E, a high-affinity ligand for lipoprotein receptors, and develops atherosclerotic plaques in a fashion similar to humans [52]. In ApoE^−/−^ mice, exposure to both ambient PM and tobacco smoke increased the plaque area in the pathological analysis of the aorta [6, 53–56]. Vascular endothelial cells were chosen as study subjects to explore molecular biology mechanisms (Cluster 5). On this topic, the induction of oxidative stress [57, 58] and mitochondrial DNA (mtDNA) damage [53, 59, 60] play a key role in the progression of atherosclerosis (Cluster 7).

## Conclusions

Increasing attention has been given to the research field of PM and atherosclerosis. The number of published articles has increased from five for the period 1973–1978 to 89 for the period 2009–2014. The characteristics of the research literature in the fields of PM and atherosclerosis can be obtained through the quantitative analysis of bibliographic information and MeSH terms. Bibliographic information includes annual publications, authors, journals, institutions and countries; which can support a deeper understanding of research directions in the future. Cluster analysis by MeSH terms can provide clues that suggest popular topics in these fields, and help determine research emphases and priorities. Based on the quantitative analysis of bibliographic information and MeSH terms, we found that “tobacco smoke pollution” and “smoke and air particulate matter” were the most studied targets in the research field of PM and atherosclerosis.

It should be pointed out that the overall characteristics of these studies might be affected to a certain extent by the interest and funding of the researchers. In conclusion, the bibliometric analysis method is an effective approach for providing the characterization of a given research field, and can be adopted by other research fields as well.

## Abbreviations

CVD: cardiovascular diseases
PM: particulate matter

## References

[1] Jia X, Guo X, Li H, An X, Zhao Y (2013). Characteristics and popular topics of latest researches into the effects of air particulate matter on cardiovascular system by bibliometric analysis. Inhal Toxicol.

[2] Pope CA, Burnett RT, Thurston GD, Thun MJ, Calle EE, Krewski D, Godleski JJ (2004). Cardiovascular mortality and long-term exposure to particulate air pollution: epidemiological evidence of general pathophysiological pathways of disease. Circulation.

[3] Sun Q, Hong X, Wold LE (2010). Cardiovascular effects of ambient particulate air pollution exposure. Circulation.

[4] Araujo JA, Barajas B, Kleinman M, Wang X, Bennett BJ, Gong KW (2008). Ambient particulate pollutants in the ultrafine range promote early atherosclerosis and systemic oxidative stress. Circ Res.

[5] Brocato J, Sun H, Shamy M, Kluz T, Alghamdi MA, Khoder MI (2014). Particulate matter from Saudi Arabia induces genes involved in inflammation, metabolic syndrome and atherosclerosis. J Toxicol Environ Health A.

[6] Chen T, Jia G, Wei Y, Li J (2013). Beijing ambient particle exposure accelerates atherosclerosis in ApoE knockout mice. Toxicol Lett.

[7] Fetterman JL, Pompilius M, Westbrook DG, Uyeminami D, Brown J, Pinkerton KE (2013). Developmental exposure to second-hand smoke increases adult atherogenesis and alters mitochondrial DNA copy number and deletions in apoE(−/−) mice. PLoS One.

[8] Fu HZ, Wang MH, Ho YS (2013). Mapping of drinking water research: a bibliometric analysis of research output during 1992–2011. Sci Total Environ.

[9] Ahmadi M, Habibi S, Sedghi S, Hosseini F (2014). Bibliometric analysis of stem cell publications in iran. Acta Inform Med.

[10] Colantonio LD, Baldridge AS, Huffman MD, Bloomfield GS, Prabhakaran D (2015). Cardiovascular research publications from Latin america between 1999 and 2008. A Bibliometric Study Arq Bras Cardiol.

[11] Mansour AM, Mollayess GE, Habib R, Arabi A, Medawar WA (2015). Bibliometric trends in ophthalmology 1997–2009. Indian J Ophthalmol.

[12] Fu C, Liu Z, Zhu F, Li S, Jiang L. A meta-analysis: is low-dose computed tomography a superior method for risky lung cancers screening population? Clin Respir J. 2014. doi:10.1111/crj.12222. 10.1111/crj.12222 25307063

[13] Ginsparg P (2011). ArXiv at 20. Nature.

[14] Sewell W (1964). Medical subject headings in medlars. Bull Med Libr Assoc.

[15] Freeman LC (1979). Centrality in social networks conceptual clarification. Soc Networks.

[16] Saade G, Seidenberg AB, Rees VW, Otrock Z, Connolly GN (2010). Indoor secondhand tobacco smoke emission levels in six Lebanese cities. Tob Control.

[17] Van Deusen A, Hyland A, Travers MJ, Wang C, Higbee C, King BA (2009). Secondhand smoke and particulate matter exposure in the home. Nicotine Tob. Res.

[18] Howard DJ, Ota RB, Briggs LA, Hampton M, Pritsos CA (1998). Environmental tobacco smoke in the workplace induces oxidative stress in employees, including increased production of 8-hydroxy-2’-deoxyguanosine. Cancer Epidemiol Biomark Prev.

[19] Kiechl S, Werner P, Egger G, Oberhollenzer F, Mayr M, Xu Q (2002). Active and passive smoking, chronic infections, and the risk of carotid atherosclerosis prospective results from the Bruneck Study. Stroke.

[20] Salonen R, Salonen JT (1991). Determinants of carotid intima‐media thickness: a population‐based ultrasonography study in eastern Finnish men. J Intern Med.

[21] Tell GS, Polak JF, Ward BJ, Kittner SJ, Savage PJ, Robbins J (1994). Relation of smoking with carotid artery wall thickness and stenosis in older adults. The cardiovascular health study. The cardiovascular health study (CHS) collaborative research group. Circulation.

[22] Willeit J, Kiechl S, Oberhollenzer F, Rungger G, Egger G, Bonora E (2000). Distinct risk profiles of early and advanced atherosclerosis prospective results from the bruneck study. Arterioscler Thromb Vasc Biol.

[23] Lu L, Mackay DF, Pell JP (2013). Association between level of exposure to secondhand smoke and peripheral arterial disease: cross-sectional study of 5,686 never smokers. Atherosclerosis.

[24] Peinemann F, Moebus S, Dragano N, Möhlenkamp S, Lehmann N, Zeeb H (2011). Secondhand smoke exposure and coronary artery calcification among nonsmoking participants of a population-based cohort. Environ Health Perspect.

[25] Prugger C, Wellmann J, Heidrich J, De Bacquer D, Perier M-C, Empana J-P (2014). Passive smoking and smoking cessation among patients with coronary heart disease across Europe: results from the EUROASPIRE III survey. Eur Heart J.

[26] Yankelevitz DF, Henschke CI, Yip R, Boffetta P, Shemesh J, Cham MD (2013). Second-hand tobacco smoke in never smokers is a significant risk factor for coronary artery calcification. J Am Coll Cardiol Img.

[27] Yuan H, Wong LS, Bhattacharya M, Ma C, Zafarani M, Yao M (2007). The effects of second-hand smoke on biological processes important in atherogenesis. BMC Cardiovasc Disord.

[28] Geerts CC, Bots ML, Grobbee DE, Uiterwaal CS (2008). Parental smoking and vascular damage in young adult offspring: is early life exposure critical? The atherosclerosis risk in young adults study. Arterioscler Thromb Vasc Biol.

[29] Kallio K, Jokinen E, Raitakari OT, Hämäläinen M, Siltala M, Volanen I (2007). Tobacco smoke exposure is associated with attenuated endothelial function in 11-year-old healthy children. Circulation.

[30] Matturri L, Ottaviani G, Corti G, Lavezzi AM (2004). Pathogenesis of early atherosclerotic lesions in infants. Pathol. Res. Pract.

[31] Benowitz NL (1999). Biomarkers of environmental tobacco smoke exposure. Environ Health Perspect.

[32] Kallio K, Jokinen E, Saarinen M, Hämäläinen M, Volanen I, Kaitosaari T (2010). Arterial intima-media thickness, endothelial function, and apolipoproteins in adolescents frequently exposed to tobacco smoke. Circ. Cardiovasc. Qual. Outcomes.

[33] Bonetti PO, Trachsel LD, Kuhn MU, Schulzki T, Erne P, Radovanovic D (2011). Incidence of acute myocardial infarction after implementation of a public smoking ban in Graubunden Switzerland: two year follow-up. Swiss Med Wkly.

[34] Herman PM, Walsh ME (2011). Hospital admissions for acute myocardial infarction, angina, stroke, and asthma after implementation of Arizona’s comprehensive statewide smoking ban. Am J Public Health.

[35] Tan CE, Glantz SA (2012). Association between smoke-free legislation and hospitalizations for cardiac, cerebrovascular, and respiratory diseases a meta-analysis. Circulation.

[36] Adar SD, Sheppard L, Vedal S, Polak JF, Sampson PD, Diez Roux AV (2013). Fine particulate air pollution and the progression of carotid intima-medial thickness: a prospective cohort study from the multi-ethnic study of atherosclerosis and air pollution. PLoS Med.

[37] Bauer M, Moebus S, Möhlenkamp S, Dragano N, Nonnemacher M, Fuchsluger M (2010). Urban particulate matter air pollution is associated with subclinical atherosclerosis: results from the HNR (Heinz Nixdorf Recall) study. J Am Coll Cardiol.

[38] Sun M, Kaufman JD, Kim SY, Larson TV, Gould TR, Polak JF (2013). Particulate matter components and subclinical atherosclerosis: common approaches to estimating exposure in a multi-ethnic study of atherosclerosis cross-sectional study. Environ Health.

[39] Tonne C, Yanosky JD, Beevers S, Wilkinson P, Kelly FJ (2012). PM mass concentration and PM oxidative potential in relation to carotid intima-media thickness. Epidemiology.

[40] O’Neill MS, Diez-Roux AV, Auchincloss AH, Shen M, Lima JA, Polak JF (2011). Long-term exposure to airborne particles and arterial stiffness: the multi-ethnic study of atherosclerosis(MESA). Environ Health Perspect.

[41] Fernández-Ortiz A, Jiménez-Borreguero LJ, Peñalvo JL, Ordovás JM, Mocoroa A, Fernández-Friera L (2013). The progression and early detection of subclinical atherosclerosis (PESA) study: rationale and design. Am Heart J.

[42] Allen RW, Adar SD, Avol E, Cohen M, Curl CL, Larson T (2012). Modeling the residential infiltration of outdoor PM2. 5 in the multi-ethnic study of atherosclerosis and air pollution (MESA Air). Environ Health Perspect.

[43] Hoffmann B, Moebus S, Kröger K, Stang A, Möhlenkamp S, Dragano N (2009). Residential exposure to urban Air pollution, ankle–brachial index, and peripheral arterial disease. Epidemiology.

[44] Zanobetti A, Gold DR, Stone PH, Suh HH, Schwartz J, Coull BA (2010). Reduction in heart rate variability with traffic and air pollution in patients with coronary artery disease. Environ Health Perspect.

[45] Campen MJ, McDonald JD, Reed MD, Seagrave JC (2006). Fresh gasoline emissions, not paved road dust, alter cardiac repolarization in ApoE−/− mice. Cardiovasc Toxicol.

[46] Maresh JG, Campen MJ, Reed MD, Darrow AL, Shohet RV (2011). Hypercholesterolemia potentiates aortic endothelial response to inhaled diesel exhaust. Inhal Toxicol.

[47] Baxter CS, Ross CS, Fabian T, Borgerson JL, Shawon J, Gandhi PD (2010). Ultrafine particle exposure during fire suppression-is it an important contributory factor for coronary heart disease in firefighters?. J Occup Environ Med.

[48] Seilkop SK, Campen MJ, Lund AK, McDonald JD, Mauderly JL (2012). Identification of chemical components of combustion emissions that affect pro-atherosclerotic vascular responses in mice. Inhal Toxicol.

[49] Vesterdal LK, Folkmann JK, Jacobsen NR, Sheykhzade M, Wallin H, Loft S (2010). Pulmonary exposure to carbon black nanoparticles and vascular effects. Part. Fibre Toxicol.

[50] LaGier AJ, Manzo ND, Carll AP, Jaskot RH, Slade R, Richards JH (2008). A hyperlipidemic rabbit model provides new insights into pulmonary zinc exposure effects on cardiovascular health. Cardiovasc Toxicol.

[51] Berger A, Zareba W, Schneider A, Rückerl R, Ibald-Mulli A, Cyrys J (2006). Runs of ventricular and supraventricular tachycardia triggered by air pollution in patients with coronary heart disease. J Occup Environ Med.

[52] Reddick RL, Zhang SH, Maeda N (1994). Atherosclerosis in mice lacking apo E. Evaluation of lesional development and progression. Arterioscler Thromb Vasc Biol.

[53] Knight-Lozano CA, Young CG, Burow DL, Hu ZY, Uyeminami D, Pinkerton KE (2002). Cigarette smoke exposure and hypercholesterolemia increase mitochondrial damage in cardiovascular tissues. Circulation.

[54] Sun Q, Yue P, Deiuliis JA, Lumeng CN, Kampfrath T, Mikolaj MB (2009). Ambient air pollution exaggerates adipose inflammation and insulin resistance in a mouse model of diet-induced obesity. Circulation.

[55] Wan Q, Cui X, Shao J, Zhou F, Jia Y, Sun X (2014). Beijing ambient particle exposure accelerates atherosclerosis in ApoE knockout mice by upregulating visfatin expression. Cell Stress Chaperones.

[56] Yang Z, Harrison CM, Chuang GC, Ballinger SW (2007). The role of tobacco smoke induced mitochondrial damage in vascular dysfunction and atherosclerosis. Mutat. Res., Fundam. Mol. Mech. Mutagen.

[57] Kunitomo M, Yamaguchi Y, Kagota S, Yoshikawa N, Nakamura K, Shinozuka K (2009). Biochemical evidence of atherosclerosis progression mediated by increased oxidative stress in apolipoprotein E-deficient spontaneously hyperlipidemic mice exposed to chronic cigarette smoke. J Pharmacol Sci.

[58] Wei H, Wei D, Yi S, Zhang F, Ding W. Oxidative stress induced by urban fine particles in cultured EA. hy926 cells. Human & experimental toxicology. 2010. 10.1177/0960327110374207 20554636

[59] Ballinger SW, Patterson C, Knight-Lozano CA, Burow DL, Conklin CA, Hu Z (2002). Mitochondrial integrity and function in atherogenesis. Circulation.

[60] Yang Z, Knight CA, Mamerow MM, Vickers K, Penn A, Postlethwait EM (2004). Prenatal environmental tobacco smoke exposure promotes adult atherogenesis and mitochondrial damage in apolipoprotein E−/− mice fed a chow diet. Circulation.

